# The optimal exercise intensity for the unbiased comparison of thermoregulatory responses between groups unmatched for body size during uncompensable heat stress

**DOI:** 10.14814/phy2.13099

**Published:** 2017-03-07

**Authors:** Nicholas Ravanelli, Matthew Cramer, Pascal Imbeault, Ollie Jay

**Affiliations:** ^1^ School of Human KineticsUniversity of OttawaOttawaCanada; ^2^ Thermal Ergonomics LaboratoryFaculty of Health SciencesUniversity of SydneySydneyNew South WalesAustralia; ^3^ Institute for Exercise and Environmental MedicineTexas Health Presbyterian Hospital DallasDallasTexas; ^4^ The University of Texas Southwestern Medical CenterTexas Health Presbyterian HospitalDallasTexas; ^5^ Charles Perkins CentreUniversity of SydneySydneyNew South WalesAustralia

**Keywords:** Body morphology, core temperature, evaporation, hyperthermia, sweating

## Abstract

We sought to identify the appropriate exercise intensity for unbiased comparisons of changes in rectal temperature (ΔT_re_) and local sweat rates (LSR) between groups unmatched for body size during uncompensable heat stress. Sixteen males vastly different in body morphology were separated into two equal groups [small (SM): 65.8 ± 6.2 kg, 1.8 ± 0.1 m^2^; large (LG): 100.0 ± 13.1 kg, 2.3 ± 0.1 m^2^], but matched for sudomotor thermosensitivity (SM: 1.3 ± 0.6; LG: 1.1 ± 0.4 mg·cm^−2^·min^−1^·°C^−1^). The maximum potential for evaporation (E_max_) for each participant was assessed using an incremental humidity protocol. On separate occasions, participants then completed 60 min of cycling in a 35°C and 70% RH environment at (1) 50% of VO_2max_, (2) a heat production (H_prod_) of 520 W, (3) H_prod_ relative to mass (6 W·kg^−1^), and (4) H_prod_ relative to mass above E_max_ (3 W·kg^−1^>E_max_). E_max_ was similar between LG (347 ± 39 W, 154 ± 15 W·m^−2^) and SM (313 ± 63 W, 176 ± 34 W·m^−2^, *P *>* *0.12). ΔT_re_ was greater in SM compared to LG at 520 W (SM: 1.5 ± 0.5; LG 0.8 ± 0.3°C, *P *<* *0.001) and at 50% of VO_2max_ (SM: 1.4 ± 0.5; LG 0.9 ± 0.3°C, *P *<* *0.001). However, ΔT_re_ was similar between groups when H_prod_ was either 6 W·kg^−1^ (SM: 0.9 ± 0.3; LG 0.9 ± 0.2°C, *P *=* *0.98) and 3 W·kg^−1^>E_max_ (SM: 1.4 ± 0.5; LG 1.3 ± 0.4°C, *P *=* *0.99). LSR was similar between LG and SM irrespective of condition, suggesting maximum LSR was attained (SM: 1.10 ± 0.23; LG: 1.07 ± 0.35 mg·cm^−2^·min^−1^, *P *=* *0.50). In conclusion, systematic differences in ΔT_re_ and LSR between groups unmatched for body size during uncompensable heat stress can be avoided by a fixed H_prod_ in W·kg^−1^ or W·kg^−1^>E_max_.

## Introduction

Assessing the influence of factors such as disease (Baker 2002; Davis et al. 2010; Benda et al. 2016) and injury (Petrofsky 1992; Crandall and Davis 2010; Pritchett et al. 2015) on the physiological capacity to regulate internal body temperature during exercise in hot and humid environments inevitably requires a comparison between independent (e.g., control and experimental) groups. If these participants are morphologically dissimilar, as is often the case, selecting an exercise intensity that ensures no systematic differences in the change in core temperature and sweating due to factors associated with differences in body size and metabolic heat production (H_prod_), is vital.

A recent series of studies from our laboratory has contributed to the development of a methodological framework for studies conducted under physiologically compensable conditions (i.e., temperate and relatively dry; ~25°C, <40% RH). Specifically, for comparisons of changes in core temperature, irrespective of maximum aerobic capacity (VO_2max_) – between ~35 to 65 mL·kg^−1^·min^−1^ (Jay et al. 2011), body mass and body surface area (Cramer and Jay 2014), cycling efficiency (Jay et al. 2011; Cramer and Jay 2014) or running economy (Smoljanić et al. 2014), an exercise intensity should be chosen to elicit a fixed H_prod_ per unit total body mass (in W·kg^−1^). However, it is presently not known whether this approach is transferable to uncompensable heat stress conditions whereby independently of mass, the major determinant of heat loss capacity is the maximum rate of evaporation (E_max_), which is ultimately limited by the absolute body surface area (BSA) that can be saturated with sweat.

Indeed, in uncompensable conditions a fixed H_prod_ in W·kg^−1^ of total body mass may systematically induce greater changes in core temperature in larger individuals secondary to their lower surface area‐to‐body mass ratio. That is, the H_prod_ in W·kg^−1^ at the limit of physiological compensation will be lower in a larger person, therefore, the rate of heat storage per unit mass (and therefore theoretically their rate of rise of core temperature) will be greater at fixed levels of H_prod_ in W·kg^−1^ in uncompensable environments. It follows that a fixed H_prod_ in W·kg^−1^ of total body weight at a level above each individual's limit of physiological compensation (i.e., W·kg^−1^ >E_max_) may be the optimal method for prescribing exercise intensity for between‐group experimental designs. However, a fixed relative exercise intensity (%VO_2max_) has been traditionally favoured for such comparisons (Saltin and Hermansen 1966; Davies et al. 1976; Greenhaff 1989), whereas more recently a fixed absolute workload (and therefore absolute H_prod_) has also been recommended (Mora‐Rodriguez 2012).

It is now well established that absolute E_req_ (in W, (Gagnon et al. 2013)) and E_req_ relative to BSA (in W·m^−2^_,_ (Cramer and Jay 2014)) primarily determine whole‐body sweat rate (WBSR) and local sweat rate (LSR) in compensable conditions, respectively. In an uncompensable environment where progressive hyperthermia develops, LSR will be determined by the elevation in internal body temperature, eventually reaching a maximum (Davies 1979; Machado‐Moreira et al. 2008). However, the same maximum LSR between two people of different body sizes will theoretically lead to a greater WBSR in the larger individual.

Similar to our previous work in compensable conditions (Jay et al. 2011; Cramer and Jay 2014), the aim of this study was to identify the optimal exercise intensity to eliminate inherent bias due to biophysical factors for the comparison of time‐dependent changes in core temperature and sweating between groups of unequal body size during uncompensable heat stress. We compared the thermoregulatory responses of two groups differing greatly in body mass and BSA‐to‐mass ratio (large (LG), small (SM)) but matched for age, sex, operational parameters for the physiological control of sweating (i.e., thermosensitivity), and maximum rate of evaporation per unit BSA (i.e., E_max_) during exercise in a hot and humid (i.e., T_a_: 36°C; RH: 70%) environment. The LG and SM groups exercised at four different intensities: (1) a fixed H_prod_ per unit mass of 6 W·kg^−1^; (2) a fixed H_prod_ per unit mass above E_max_ of 3 W·kg^−1^ > E_max_; (3) a relative intensity of 50%VO_2max_; and (4) an absolute H_prod_ of 520 W. It was hypothesized that H_prod_ per unit mass at a fixed level above E_max_ (i.e., W·kg^−1^>E_max_) would yield similar changes in core temperature despite large differences in body mass and BSA‐to‐mass ratio, while systematic differences between LG and SM groups related to biophysical factors would be observed with exercise intensity prescribed at a fixed H_prod_ in W, W·kg^−1^ and %VO_2max_. It was also hypothesized that a same maximum LSR would be observed in both groups irrespective of the exercise intensity, and thus a greater WBSR in the LG group.

## Methods

### Participants

Ethical approval was obtained from the University of Ottawa Health Sciences Research Ethics Board (H12‐11‐05) conforming to the principles set forth in the Declaration of Helsinki 2013. All volunteers gave both verbal and written consent prior to any preliminary and experimental trials, and were required to fill out a Physical Activity Readiness Questionnaire and an American Heart Association Pre‐Participation Screening Questionnaire.

A power calculation with G*Power (3.1.9.2) using *α*‐ and *β*‐ values set to 0.05 and 0.95, respectively, determined that a sample size of 16 subjects (eight per group) was required to report a significant difference between ΔT_re_ in two groups different in mass (~20 kg) following 60 min of exercise at 500 W of H_prod_ with a mean between‐group difference of 0.5°C and a standard deviation of 0.2°C (Cramer and Jay 2014). A total of sixteen men separated equally into two groups (8 large, LG; 8 small, SM) with a mean difference in body mass and BSA (estimated using the DuBois & DuBois equation (1916)) of ~30 kg and ~0.4 m^2^, respectively (Table 1), participated in the study. Groups were matched for age, but not aerobic fitness to ensure differences in %VO_2max_ in trials with H_prod_ divisible by total body mass.

**Table 1:** Mean participant physical characteristics

	Age (years)	Mass (kg)	BSA (m^2^)	BSA/mass (cm^2^·kg^−1^)	Body fat (%)	VO_2max_ (ml·kg^−1^·min^−1^)
SM	25 ± 5	65.8 ± 6.2	1.8 ± 0.1	271 ± 17	12.3 ± 3.5	54.7 ± 4.6^a^
LG	25 ± 3	100.0 ± 13.1^a^	2.3 ± 0.1^a^	226 ± 17^a^	24.9 ± 8.2^a^	38.5 ± 9.0

LG, large body size group; SM, small body size group; BSA, body surface area; VO_2max_ maximum rate of oxygen uptake.

a Significant difference (*P *<* *0.05).

### Preliminary session

Participants performed a preliminary session during which anthropometry and maximal aerobic capacity were assessed. Height and weight were also measured using a wall‐mounted stadiometer (HR‐200, Tanita, Arlington Heights, IL) and digital scale (BWB‐800, Tanita, Arlington Heights, IL), respectively. Body composition was measured by dual‐energy x‐ray absorptiometry (GE‐LUNAR Prodigy module, GE Medical Systems, Madison, WI). Aerobic fitness (VO_2max_) was assessed using an incremental exercise test to exhaustion on an upright cycle ergometer (Kettler ErgoRace, Virginia Beach, VA) in accordance with guidelines from the Canadian Society of Exercise Physiology (CSEP, 1996). Following a self‐paced warmup and at least 10‐minute rest period, the protocol commenced with an external workload of 80 W that increased at a rate of 20 W·min^−1^ until physical or volitional exhaustion. Expired gases were measured via breath‐by‐breath indirect calorimetry using a metabolic cart (Vmax Encore, Care Fusion, Yorba Linda, CA).

### Experimental design

Prior to all experimental sessions, participants were asked to abstain from alcohol, caffeine, and strenuous exercise for at least 12 h. In addition, they were asked to eat a light meal and drink ~500 mL of water 2 h before arrival. Experimental trials were conducted at the same time of day and separated by 48 h to eliminate any influence of circadian variation. Participants first completed the E_max_ assessment (described below) followed by the remaining four experimental trials in a counter‐balanced order (i. 50% of VO_2max_; ii. fixed H_prod_ of 520 W; iii. fixed H_prod_ of 6 W·kg^−1^; iv. fixed H_prod_ of 3 W·kg^−1^>E_max_).

### Instrumentation

Ambient temperature and absolute humidity were measured using a dew point mirror (473 RH Systems, Albuquerque, NM). Rectal temperature (T_re_) and oesophageal temperature (T_es_) were measured using paediatric grade thermistor probes (Mon‐a‐therm^®^, Mallinckrodt Medical, St. Louis, MO). The T_re_ probe was inserted to a depth of 20 cm past the anal sphincter and the T_es_ probe was inserted 40 cm through the nasal cavity into the oesophagus, estimated to be the region close to the left ventricle (Mekjavic and Rempel 1990). Four surface thermistors (Concept Engineering, Old Saybrook, CT) were affixed to the skin using surgical tape (Transpore^®^, 3M, London, ON). Mean skin temperature (T_sk_) was calculated using the Ramanathan weighting coefficients (Ramanathan 1964): chest 30%, triceps 30%, thigh 20%, and calf 20%. All thermometric measures were sampled every 5 sec (NI cDAQ‐91722 module, National Instruments, Austin, TX) and displayed in real‐time on a desktop computer using customized LabView software (v7.0, National Instruments, Austin, TX).

Local sweat rates (LSR) of the upper back (inferior to the scapular spine and ~5 cm from the axilla) and forearm (midpoint of the anterior distal segment) were measured using ventilated sweat capsules. Anhydrous air was supplied to each 4.1‐cm^2^ capsule at a continuous flow rate of 1.00 L min^−1^ and 0.83 L min^−1^ for back and forearm, respectively (Omega FMA‐A2307, Omega Engineering, Stamford, CT). Capsules were secured to the skin using skin glue (Collodion USP MD0002, Mavidon, Lake Worth, FL) and additional surgical tape. The temperature and humidity of outflowing air from the capsules were measured every 5 sec using factory‐calibrated capacitance hygrometers (HMT333, Vaisala, Vantaa, Finland). Local sweat rate of the back and forearm were then calculated as the product of absolute humidity and flow rate, and expressed relative to the area under the capsule in milligrams per square centimetre per minute (mg·cm^−2^·min^−1^) and averaged between sites (LSR_mean_). Sudomotor thermosensitivity was determined for each individual trial using linear regression of 1‐min averages of the change in mean body temperature (ΔT_b_) calculated as a weighted average between T_es_ (80%) and T_sk_ (20%) (Vieth 1989; Cheuvront et al. 2009) with LSR_mean_.

### Protocol

Participants provided a urine sample immediately prior to all experimental trials. A refractometer (Reichert TS 400, Depew, NY) measured urine specific gravity (USG) and the cut‐off value of greater than 1.025 was used to ensure pre‐exercise euhydration (Kenefick and Cheuvront 2012). Participants where then given a pair of standardized running shorts and non‐absorbent sandals to wear and inserted the T_re_ probe. Next, an initial body mass measurement was taken which was used to calculate the appropriate H_prod_ for exercise intensities fixed relative to body mass. Prior to entering the climatic chamber, a T_es_ probe was inserted and the two ventilated sweat capsules were affixed to the skin. Participants then entered the climate chamber where the remaining instrumentation was completed. Thirty minutes of rest then followed to equilibrate with the environment.

#### Experimental trial 1 (E_max_ Assessment)

**Figure 1:**
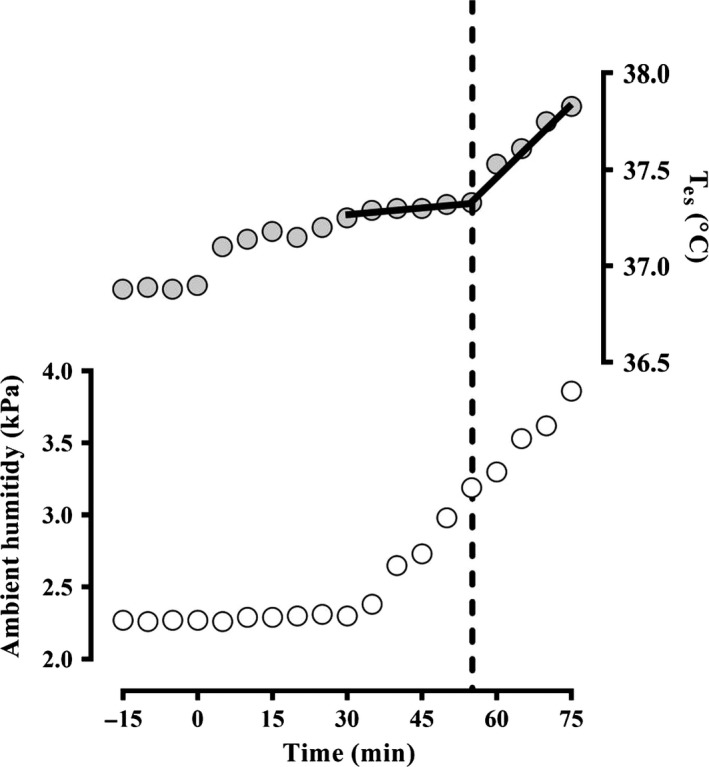
Example of method used to determine *K* coefficient from E_max_ assessment trial using segmental linear regression to assess the upward rise in oesophageal temperature (T_es_). The slope of first segment was constricted such that it did not exceed a rate of change in T_es_ equivalent to 0.1°C·15 min^−1^. Heat balance parameters coinciding with the point of inflection are used to derive K (Equation 8).

We used an incremental humidity protocol first described by Kamon and Belding (1971) and subsequently revised and refined first by Kamon and Avellini (1976, 1979) and later by Kenney et al. (1993), Kenney and Zeman (2002), and Dougherty et al. (2009) to minimize trial duration and the number of tests required. The climate chamber was initially maintained at baseline conditions of 36.1 ± 0.3°C and 39.0 ± 1.8%RH (2.3 ± 0.1 kPa) with a fixed air velocity of 1.2 m·s^−1^. Participants began exercising on an upright cycle ergometer at a fixed external work rate of 100 W. After 30 min of exercise by which time a steady‐state core temperature (and therefore presumably heat balance) had been reached, ambient vapour pressure was increased at a rate of 0.3 kPa (~5%RH) every 7.5 min in a stepwise fashion for up to 45 min, while ambient temperature remained fixed. E_max_ was derived using the absolute humidity at which an upward inflection in T_es_ was observed (Fig. 1) indicating a transition from a compensable (defined as a rate of rise in T_es_ of 0.1°C·15 min^−1^) to an uncompensable condition. To verify this transition, participants continued to cycle for at least another 10 min (while ambient vapour pressure continued to increase) following the inflection of T_es_ to ensure T_es_ continued to rise. The critical absolute humidity at which this T_es_ inflection occurred was then objectively determined using segmental linear regression (Ravanelli et al. 2015) from the 30th min of exercise. One participant in the LG group was unable to insert a T_es_ thermistor, thus, his E_max_ was assumed to be equal to the mean of the LG group.

#### Experimental trials 2 to 5

For all remaining experimental trials, the environmental conditions were maintained at 36.2 ± 0.2°C and 69.7 ± 1.3%RH throughout. Following 30 min of rest, participants began exercising at one of the four predetermined exercise intensities for up to 75 min. All exercise sessions were at least 45 min; with early termination due to either volitional exhaustion (*n* = 12 of 64 trials) or T_re_ exceeding 39.5°C (*n* = 2 of 64 trials). The minimum exercise duration for each condition was 50 min for 50%VO_2max_ (SM: 70.6 ± 8.2 min, LG: 68.4 ± 9.4 min), 55 min for 520 W (SM: 68.8 ± 8.8 min, LG: 68.4 ± 9.4 min), and 45 mins for both 6 W·kg^−1^ (SM: 75 ± 0 min, LG: 67.5 ± 11.3 min) and 3 W·kg^−1^ > E_max_ (SM: 73.1 ± 5.3 min, LG: 65.6 ± 13.7 min). By design, some exercise intensities were equal to other conditions for some participants (i.e., 520 W was the equivalent of 6 W·kg^−1^ for LG). Core temperature (T_re_), mean skin temperature (T_sk_), and LSR were measured throughout the trial, while T_es_ was only measured for first 20 min of exercise to determine sudomotor thermosensitivity. Immediately before and after exercise, nude (unclothed but instrumented) body mass was measured in triplicate using a balance scale (Combics 2, Sartorius, Mississauga, ON, Canada); instrument wires were taped to an adjacent stand in an identical way for all measurements. The difference between pre‐ and post body mass (assumed to be total sweat loss) was divided by the time elapsed between the two measurements and expressed as WBSR in g·h^−1^.

### Calculations

The evaporative requirement to maintain heat balance (E_req_) in W·m^−2^ was estimated by rearranging the conceptual heat balance equation:

(1) $$E_{\mathrm{req}}=H_{\mathrm{prod}}-\left(H_{\mathrm{dry}}+H_{\mathrm{res}}\right)\left[W\cdot m^{-2}\right]$$

The rate of metabolic heat production (H_prod_) was calculated by subtracting the rate of external work regulated by the cycle ergometer (in W) from metabolic energy expenditure (M). M was estimated using the following equation (Nishi 1981):

(2) $$M=\dot{V}O_{2}\frac{\left(\left(\frac{\mathrm{RER}-0.7}{0.3}\right)e_{c}\right)+\left(\left(\frac{1.0-\mathrm{RER}}{0.3}\right)e_{f}\right)}{60\cdot A_{D}}\cdot 1000\;\left[W\cdot m^{-2}\right]$$

Where: $\dot{V}$O_2_ is the rate of oxygen consumption (L/min), e_c_ is the caloric equivalent per litre of oxygen for the oxidation of carbohydrates (21.13 kJ per L of O_2_ consumed), e_f_ is the caloric equivalent per litre of oxygen for the oxidation of lipids (19.62 kJ per L of O_2_ consumed), and respiratory exchange ratio (RER) is the ratio of carbon dioxide production and oxygen consumption (VCO_2_/VO_2_).

The rate of dry heat loss (H_dry_) via convection and radiation is primarily governed by the temperature gradient between skin (T_sk_) and air (T_a_) and mean radiant (T_r_) temperature, respectively. By design, the conditions of this study were selected to ensure a very small T_sk_‐T_a_/T_r_ gradient (i.e., T_a_ ≈ T_sk_; and assuming T_a_ = T_r_) so that absolute error associated with estimating dry clothing insulation and whole‐body air velocity was minimized. Nevertheless, even though H_dry_ was not greater than 15 W·m^−2^ at any point for any participant, values were still calculated using the standard approach detailed in the literature (Parsons 2002).

The rate of respiratory heat loss (H_res_) was estimated using the following:

(3) $$H_{\mathrm{res}}=0.0173\cdot \left(H_{\mathrm{prod}}\right)\cdot \left(5.87-P_{a}\right)+0.0014\cdot \left(H_{\mathrm{prod}}\right)\cdot \left(34-T_{a}\right)\left[W\cdot m^{-2}\right]$$

Where: P_a_ was the ambient vapour pressure (in kPa), and T_a_ was the ambient temperature (in °C).

### Determining E_max_ (from Experimental trial 1)

The maximum rate of evaporation (E_max_) is equal to:

(4) $$E_{\max}=\omega_{\max}\left(P_{\mathrm{sk},s}-P_{a}\right)/\left(R_{e,\mathrm{cl}}+\left[1/h_{e}*f_{\mathrm{cl}}\right]\right)\left[W\cdot m^{-2}\right]$$

Where: *ω*_max_ is the maximum skin wettedness (Gagge 1937), which can theoretically range from 0.85 (or lower) to 1.00 (Candas et al. 1979b); P_a_ is the absolute ambient vapour pressure at E_max_ (in kPa), which is equal to P_crit_ measured in experimental trial 1 (Fig. 1). P_sk,s_ (in kPa) was the saturated water vapour pressure at skin temperature and was derived using Antoine's equation:

(5) $$P_{\mathrm{sk},s}=(\text{exp}\left(18.956-\left[4030.18/\left(T_{\mathrm{sk}}+235\right)\right]\right)/10\left[\text{kPa}\right]$$

Where: T_sk_ is mean skin temperature (°C).

R_e,cl_ is the evaporative heat transfer resistance of the clothing ensemble in kPa·m^2^·W^−1^, which must be measured using a sweating thermal manikin or estimated from standardized tables (Oohori et al. 1984; Parsons 2002); f_cl_ is the clothing area factor (surface area of the clothed body divided by the surface area of the nude body; ND), which is estimated using the dry heat transfer resistance (Holmér et al. 1999; Parsons et al. 1999; Parsons 2002), which itself must be either measured using a hot plate or manikin, or estimated from tables; and h_e_ is the evaporative heat transfer coefficient in W·m^−2^·kPa^−1^ that is derived directly from the convective heat transfer coefficient which itself is dependent on an accurate measurement of whole‐body air velocity.

To overcome these substantial limitations we defined E_max_ for each participant using a humidity ramp protocol in Experimental trial 1. The boundary of compensability is, by definition, the point at which E_max_ is equal to E_req_. Thus, at the critical ambient vapour pressure point at which an inflection in T_es_ was observed (P_crit_; Fig. 1), E_max_ can be substituted for E_req_, therefore:

(6) $$E_{\mathrm{req}}=\omega_{\max}\left(P_{s,\mathrm{sk}}-P_{\mathrm{crit}}\right)/\left(R_{e,\mathrm{cl}}+\left[1/h_{e}*f_{\mathrm{cl}}\right]\right)\left[W\cdot m^{-2}\right]$$

While one could estimate or measure *ω*_max_, R_e,cl_, h_e_, and f_cl,_ any inaccuracies may be amplified. However, *ω*_max_, R_e,cl_, h_e_, and f_cl_ can be combined into a single coefficient (*K*) for estimating E_max_ for our fixed experimental conditions, giving:

(7) $$E_{\mathrm{req}}=K\left(P_{\mathrm{sk},s}-P_{\mathrm{crit}}\right)$$

And K can be derived for each individual using three directly measured variables from Experimental trial 1:

(8) $$K=E_{\mathrm{req}}/\left(P_{\mathrm{sk},s}-P_{\mathrm{crit}}\right)\left[\text{ND}\right]$$

Each individual K value (which was a combined term incorporating individual's *ω*_max_, R_e,cl_, h_e_, and f_cl_ values) was then used to determine their predicted individual E_max_ value under the fixed environmental conditions (36°C, 70% RH with identical air velocity, clothing, and exercise mode to trial 1) in Experimental trials 2 to 5, using:

(9) $$E_{\max}=K\left(5.60-4.16\right)$$

Where: 5.60 (kPa) is the saturated water vapour pressure at the anticipated T_sk_ based on the inflection trial (35°C; (Alber‐Wallerström 1985)); and 4.16 (kPa) is the ambient vapour pressure (70% RH at 36°C). The E_max_ value was then converted from W·m^−2^ to W·kg^−1^, and the H_prod_ for the 3 W·kg^−1^>E_max_ trial for each individual was determined.

### Statistical analysis

All data are expressed as a mean with standard deviation (mean ± SD). Independent samples t‐tests compared SM and LG for participant characteristics, K, E_max_, H_prod_, %VO_2max_, WBSR, and sudomotor thermosensitivities. Two‐way mixed analyses of variance (ANOVA) were used to compare 1‐min averages of ΔT_re_, ΔT_sk_, and LSR with the repeated factor of time (7 levels: 0, 10, 20, 30, 40, 50, and 60 min) and the nonrepeated factor of body size (two levels: SM and LG) for experimental trials 2‐5. In the case of a significant interaction, differences between groups were assessed using independent sample t‐tests with a Holm‐Bonferroni correction. All statistical analyses were conducted using GraphPad Prism Version 6.0 for Windows (Graphpad Software, La Jolla, CA).

## Results

### Participant characteristics

By design, a greater body mass (*P* < 0.001) and BSA (*P* < 0.001) were observed in the LG group (Table 1), whereas, a higher VO_2max_ (*P* = 0.001), BSA‐to‐mass ratio (*P *<* *0.001), and lower body fat percentage (*P *=* *0.003) were observed in the SM group (Table 1). No differences in USG (*P *=* *0.93) were observed between SM (1.013 ± 0.006) and LG (1.012 ± 0.007) prior to all experimental sessions.

### E_max_ assessment

The incremental humidity protocol in experimental trial 1 yielded similar P_crit_ values between groups (SM: 3.18 ± 0.35 kPa; LG: 3.00 ± 0.30 kPa, *P *=* *0.33), and thus similar (*P *=* *0.12) derived K coefficient values (Table 2), which were then utilized to derive E_max_ values under the fixed environmental conditions (36°C, 70% RH) in experimental trials 2‐5. These estimated E_max_ values (Table 2) were similar when expressed in absolute terms (i.e., in W; *P *=* *0.22) and relative to surface area (i.e., in W·m^−2^; *P *=* *0.12). However as expected, due to differences in BSA‐to‐mass ratio between groups lower E_max_ values were observed in the LG group when expressed relative to body mass (i.e., in W·kg^−1^; *P *=* *0.006).

**Table 2:** Mean E_max_ assessment characteristics

		E_max_ at 36°C 70% RH		
	*K* coefficient	W	W·m^−2^	W·kg^−1^
SM	126.0 ± 24.3	322 ± 65	181 ± 34	4.9 ± 1.1*
LG	110.3 ± 11.1	357 ± 40	158 ± 15	3.6 ± 0.4

**LG**, large body morphology group; **SM**, small body morphology group; **E**_**max,**_ Maximum evaporative potential. Significantly greater than LG group (*P *<* *0.05).

### Core and skin temperatures

**Figure 2:**
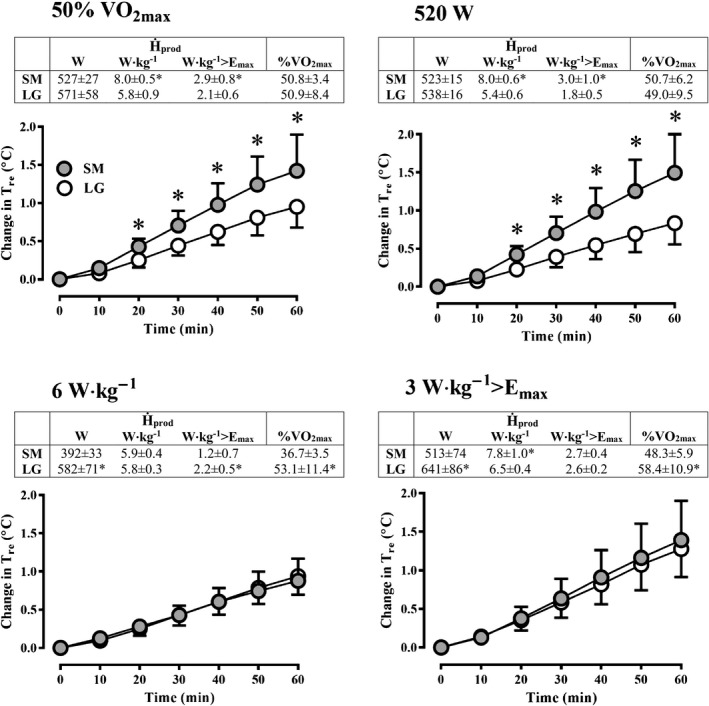
The mean change in rectal temperature (T_re_) of the small (SM) and large (LG) group over time during exercise at 50%VO_2max_ (top‐left), 520 W of heat production (H_prod_; top‐right), 6 W·kg^−1^ (bottom‐left), and 3 W·kg^−1^>E_max_ (bottom‐right). The table above each panel displays mean H_prod_ expressed in absolute W, relative to body mass (W·kg^−1^), relative to body mass above maximum evaporative potential (E_max_), and %VO_2max_. *Significant difference (*P *<* *0.05).

**Figure 3:**
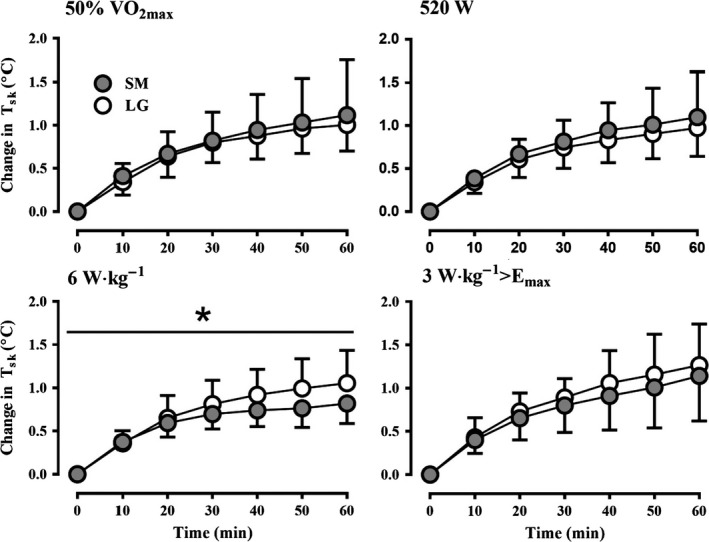
The mean change in skin temperature (T_sk_) of the small (SM) and large (LG) group over time during exercise at 50%VO_2max_ (top‐left), 520 W H_prod_ (top‐right), 6 W·kg^−1^ H_prod_ (bottom‐left), and 3 W·kg^−1^ H_prod_>E_max_ (bottom‐right). *Significant interaction (*P *=* *0.01).

The change in T_re_ (Fig. 2) was greater from 20 min onwards in the SM compared to LG group at both 50% of VO_2max_ (*P* < 0.001) and 520 W absolute H_prod_ (*P* < 0.001). In parallel, H_prod_ in W·kg^−1^ and W·kg^−1^>E_max_ was greater in the SM compared to the LG group at 50% of VO_2max_ (*P* < 0.05) and 520 W of H_prod_ (*P* < 0.05). In contrast, no differences were observed for the change in T_re_ between SM and LG at a H_prod_ of 6 W·kg^−1^ (*P* = 0.88) or 3 W·kg^−1^>E_max_ (*P* = 0.92). In addition, the change in T_sk_ was greater over time in LG compared to SM at a H_prod_ of 6 W·kg^−1^ (*P* < 0.05), while all other exercise intensities (50% VO_2max_, 520 W, and 3 W·kg^−1^>E_max_) yielded similar changes in T_sk_ between groups (Fig. 3).

### Whole‐body sweating

WBSR was similar between groups at 50% VO_2max_ (SM: 903 ± 362 g·h^−1^; LG: 855 ± 174 g·h^−1^, *P *=* *0.74), 520 W (SM: 890 ± 368 g·h^−1^; LG: 857 ± 175 g·h^−1^, *P *=* *0.82), 6 W·kg^−1^ (SM: 713 ± 192 g·h^−1^; LG: 871 ± 208 g·h^−1^, *P *=* *0.14), or 3 W·kg^−1^>E_max_ (SM: 872 ± 362 g·h^−1^; LG: 892 ± 210 g·h^−1^, *P *=* *0.89). Despite similar WBSR, E_req_ (in W) was greater in LG compared to SM at 50% VO_2max_ (SM: 531 ± 26 W; LG: 580 ± 54 W, *P *=* *0.04), 6 W·kg^−1^ (SM: 408 ± 34 W; LG: 594 ± 65 W, *P *<* *0.001), and 3 W·kg^−1^>E_max_ (SM: 519 ± 67 g·h^−1^; LG: 642 ± 78, *P *<* *0.001). By design, E_req_ was similar between SM and LG at 520 W (SM: 535 ± 16 W; LG: 548 ± 11 W, *P *=* *0.12).

### Local sweating

LSR was greater at the onset of exercise in LG compared to SM at 520 W (SM: 0.29 ± 0.07 mg·cm^−2^·min^−1^; LG: 0.49 ± 0.22 mg·cm^−2^·min^−1^, *P* = 0.03), with a trend for a greater LSR observed in LG at 50% of VO_2max_ (SM: 0.30 ± 0.07 mg·cm^−2^·min^−1^; LG: 0.46 ± 0.23 mg·cm^−2^·min^−1^, *P* = 0.08), 6 W·kg^−1^ (SM: 0.32 ± 0.08 mg·cm^−2^·min^−1^; LG: 0.48 ± 0.22 mg·cm^−2^·min^−1^, *P* = 0.08), and 3 W·kg^−1^>E_max_ (SM: 0.32 ± 0.06 mg·cm^−2^·min^−1^; LG: 0.45 ± 0.23 mg·cm^−2^·min^−1^, *P* = 0.10). However, LSR from 10 min onwards was similar between groups at 50% VO_2max_ (SM: 1.08 ± 0.28 mg·cm^−2^·min^−1^; LG: 1.09 ±0.36 mg·cm^−2^·min^−1^, *P* = 0.97), 520 W (SM: 1.07 ±0.29 mg·cm^−2^·min^−1^; LG: 1.05 ± 0.38, *P* = 0.95), 6 W·kg^−1^ (SM: 1.03 ± 0.20 mg·cm^−2^·min^−1^; LG: 1.03 ±0.39 mg·cm^−2^·min^−1^, *P* = 0.99), and 3 W·kg^−1^ > E_max_ (SM: 1.21 ± 0.16 mg·cm^−2^·min^−1^; LG: 1.10 ± 0.30mg·cm^−2^·min^−1^, *P* = 0.38). A similar LSR between LG and SM for both the forearm and upper back was observed for each condition. Meanwhile, values for E_req_ (in W·m^−2^; relative to BSA) were greater in SM at 520 W (SM: 296 ± 13 W·m^−2^; LG: 246 ± 13 W·m^−2^, *P* < 0.001), 50%VO_2max_ (SM: 299 ± 12 W·m^−2^; LG: 259 ± 29 W·m^−2^, *P* = 0.003), and 6 W·kg^−1^ (SM: 229 ± 14 W·m^−2^; LG: 265 ± 19 W·m^−2^, *P* = 0.001), while a similar E_req_ was observed between groups at 3 W·kg^−1^>E_max_ (SM: 292 ± 32 W·m^−2^; LG: 286 ± 23 W·m^−2^, *P* = 0.69).

### LSR‐T_b_ sensitivities

LSR‐T_b_ sensitivity (Table 3) was similar between LG and SM at 50% VO_2max_ (*P* = 0.65), 520 W (*P* = 0.51), 6 W·kg^−1^ (*P* = 0.20) and 3 W·kg^−1^ > E_max_ (*P* = 0.51).

**Table 3:** Mean LSR thermosensitivity for LG and SM at each condition

	Thermosensitivity (mg·cm^−2^·min^−1^·°C^−1^)			
	50% VO_2max_	520 W	6 W·kg^−1^	3 W·kg^−1^>E_max_
*SM*	1.3 ± 0.6	1.3 ± 0.6	1.4 ± 0.4	1.3 ± 06
*LG*	1.2 ± 0.4	1.1 ± 0.3	1.1 ± 0.3	1.1 ± 0.4

**LG**, large body morphology group; **SM**, small body morphology group; **E**_**max,**_ Maximum evaporative potential.

## Discussion

This study demonstrates that in an uncompensable environment, large differences in body size independently leads to systematically different changes in core temperature during exercise at a fixed absolute H_prod_ in W. On the other hand, when exercise intensity is set to elicit the same H_prod_ in W·kg^−1^ of total body mass, any systematic difference in core temperature between LG and SM is eliminated; however, greater changes T_sk_ are observed in larger individuals. If exercise is conducted at a fixed H_prod_ in W·kg^−1^>E_max_, differences in both core temperature and T_sk_ between LG and SM are abolished. Exercise at a fixed 50% VO_2max_ resulted in much greater changes in core temperature in the SM group, as their H_prod_ per unit mass was greater secondary to their different VO_2max_, which was higher in this study by design. Absolute E_req_ was similar at 520 W and different at all other intensities, while E_req_ in W·m^−2^ was only the same at 3 W·kg^−1^>E_max_. However, WBSR and LSR were similar between LG and SM at all intensities indicating maximum sweat rates were attained regardless of the uncompensable heat stress imposed. Collectively, the present data demonstrate that the methodological framework previously proposed by our group for performing unbiased comparisons of core temperature changes between independent groups in compensable conditions is largely transferable to uncompensable environments. However, for the assessment of local and whole‐body sweating responses, our data indicate that once maximum sweat rates are reached the influence of the exercise intensity method used may be indistinguishable.

### Core temperature

Although prescribing exercise intensity relative to an individual's VO_2max_ has been historically thought to normalize the putative effect of aerobic fitness on the exercise core temperature response (Saltin and Hermansen 1966; Davies et al. 1976; Greenhaff 1989), this approach does not yield similar changes in core temperature between groups differing in VO_2max_ during compensable heat stress when eliminating differences in body mass during cycle ergometry (Cramer and Jay 2014) and treadmill running (Smoljanić et al. 2014). For a given %VO_2max_, an aerobically fit individual will inevitably work at a greater H_prod_ per unit mass in comparison to an unfit person. Therefore, a greater change in core temperature should be observed in fitter individuals independently of body size (Mora‐Rodriguez et al. 2010; Cramer et al. 2012). In this study, a greater rise in T_re_ was observed in the fitter SM group (Fig. 2) in the 50% VO_2max_ trial in parallel to a H_prod_ that was >2 W·kg^−1^ higher than the LG group. Meanwhile, when %VO_2max_ was different between groups in the 6 W·kg^−1^ and 3 W·kg^−1^>E_max_ trials, the rise in T_re_ was similar (Fig. 2). Taken together, these data further demonstrate that the use of a fixed relative intensity is unsuitable for assessing differences in core temperature changes between groups in an uncompensable environment.

Recent work from our laboratory has demonstrated that in compensable conditions, using a fixed H_prod_ in W·kg^−1^ of total body mass eliminates the systematic difference in ΔT_re_ observed at a fixed absolute H_prod_ in W between groups of different body sizes (Cramer and Jay 2014). In this study, the same systematic difference between LG and SM was expected and observed (Fig. 2) at a H_prod_ of 520 W. However, it was hypothesized that the utility of a fixed H_prod_ in W·kg^−1^ for fully eliminating systematic differences in ΔT_re_ may not fully translate to uncompensable conditions. In theory, even with a similar E_max_ in W·m^−2^ (Table 2), larger individuals who invariably have a lower BSA/mass ratio will have a lower E_max_ when expressed in W·kg^−1^. It follows that the H_prod_ in W·kg^−1^ needed to exceed E_max_ will thereby be lower in the LG group (Table 2). Thus, in an uncompensable environment a fixed H_prod_ in W·kg^−1^ would be expected to be *more* uncompensable (i.e., the gap between H_prod_ and E_max_ in W·kg^−1^ is wider) for a larger individual and therefore elicit a greater ΔT_re_ compared to a smaller person. It was therefore proposed that to account for this biophysical disparity in the degree of uncompensability between different body sizes exercise should be prescribed to elicit a fixed H_prod_ per unit mass *above* each individual's E_max_ (i.e., W·kg^−1^ >E_max_). However, a similar ΔT_re_ was observed between LG and SM after 60 min of exercise in both the 6 W·kg^−1^ trial and the 3 W·kg^−1^>E_max_ trial (Fig. 2). These similar ΔT_re_ responses may be explained by the rather small difference in H_prod_ above E_max_ (~1 in W·kg^−1^ >E_max_) between both groups despite a 45 cm^2^·kg^−1^ difference in BSA/mass ratio in the 6 W·kg^−1^ trial. Nevertheless, a smaller ΔT_sk_ was observed in SM group in the 6 W·kg^−1^ condition (Fig. 3). Thus, to ensure no systematic bias when comparing changes in *both* core temperature and mean skin temperature during uncompensable heat stress, it is suggested that an exercise intensity that elicits a fixed H_prod_ above an individual's estimated E_max_ should be utilized. However, if changes in core temperature are the primary focus of a particular study, a fixed H_prod_ in W·kg^−1^ of total body mass can also be recommended. This latter approach is more straightforward as it does not require the somewhat complicated estimation (or measurement) of E_max_ for each individual.

The similar core temperature responses at fixed rates of heat production normalized for body mass between groups differing greatly in body size were observed despite a greater body fatness in the LG group (~25% vs. ~12%; Table 1). If body fatness provided an insulation effect, a greater rise in core temperature would have been expected in the LG group. Selkirk and McLellan (2001) reported a greater rise in core temperature in a trained higher adiposity group relative to a similarly trained lower adiposity group (~19% vs. ~11% body fat) with an ~10 kg smaller body mass from 40 min onwards during exercise at a similar heat production in W·kg^−1^. These opposing observations between studies may be due to stark differences in clothing. The participants in Selkirk and McLellan (2001) were likely closer to adiabatic in a semi impermeable protective ensemble than the semi‐nude participants in this study. In a scenario with zero heat dissipation from the skin to the surrounding environment, it is possible that a lower mean specific heat capacity of the body, associated with greater body fatness, may exert a greater influence on the rise in core temperature.

### Sweating

In order to identify the independent influence of body morphology on the time‐dependent changes in thermoregulatory responses in an uncompensable environment it was ensured that LG and SM groups were similar in terms of their physiological control of sudomotor activity (i.e., thermosensitivity) and maximum capacity for evaporative heat loss when normalized to BSA (i.e., E_max_ in W·m^−2^). However, as hypothesized E_max_ was greater in the SM group when expressed relative to mass (W·kg^−1^) due to their greater BSA/mass ratio.

Under compensable conditions with 100% sweating efficiency, absolute E_req_ (in W) determines WBSR (Jay et al. 2011; Gagnon et al. 2013), and E_req_ (in W·m^−2^) primarily determines LSR (Cramer and Jay 2014), with some potential modification from very large differences in BSA/mass ratio (Notley et al. 2016). However, as the skin wettedness required for heat balance (i.e., proportion of BSA that must be saturated in sweat) increases, sweat efficiency declines (i.e., more sweat drips off the body) as a result of greater sweat rates for the attainment of heat balance (Candas et al. 1979a; Alber‐Wallerström 1985). But, in an uncompensable heat stress situation once E_req_ exceeds E_max_ the rate of evaporative heat loss is essentially fixed even with different sweat rates. Nevertheless, greater sweating would still be expected with greater internal temperatures. We observed a similar WBSR and LSR between both groups in all conditions despite differences in E_req_ (in W and W·m^−2^) and ΔT_re_ in most conditions, which suggest maximum local sweat rates were attained. Theoretically, a similar LSR measured over a fixed surface area between two individuals differing greatly in BSA should result in a greater absolute WBSR (in L·h^−1^) in the larger individual as previously shown in compensable conditions with 100% sweating efficiency (Cramer and Jay 2014). The lack of dissociation between LSR and WBSR in this study is likely explained by the measurement methods and what they specifically represent; ventilated sweat capsules operate on the principle that complete evaporation occurs over a small surface area under the capsule, whereas the evaporation of sweat for a WBSR measurement is directly dependent on the ambient environment. Thus, decrements in sweating efficiency will not be observed under a capsule, even if sweating efficiency is greatly reduced over the rest of the body. In support, Gonzalez et al. (1974) demonstrated a progressive rise in core temperature and LSR with a ventilated sweat capsule, alongside an opposing decline in WBSR measured with continuous weighing, as sweating efficiency gradually reduced during an uncompensable heat stress. They and others (Peiss et al. 1956; Collins and Weiner 1962; Candas et al. 1983) have suggested excess saturation of the skin with sweat, as commonly observed in uncompensable conditions, suppresses sweating via mechanical obstruction lending to a reduced total sweat loss. Thus, the similar WBSR between SM and LG in this study may be associated with these previously reported phenomena, however, further evidence is required. Nevertheless, the similar LSR between both groups irrespective of exercise intensity demonstrates that matching for body size may not be required for unbiased comparisons of time‐dependent changes in LSR during uncompensable heat stress.

### Perspectives

**Figure 4:**
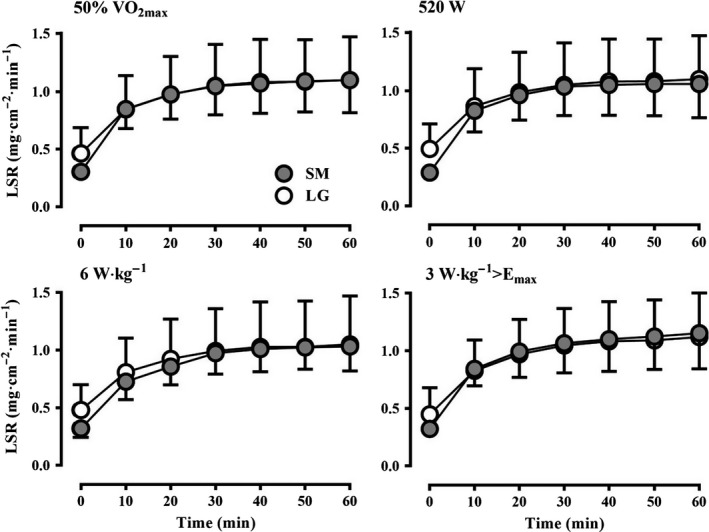
Mean local sweat rate (LSR) of the small (SM) and large (LG) group at 50%VO_2max_ (top‐left), 520 W H_prod_ (top‐right), 6 W·kg^−1^ H_prod_ (bottom‐left), and 3 W·kg^−1^ H_prod_>E_max_ (bottom‐right).

This study expands our previous work and further demonstrates the importance of accounting for biophysical factors when comparing time‐dependent changes in core temperature and sweating between groups unmatched for body size but with similar sudomotor function (i.e., thermosensitivity) during uncompensable heat stress. In contrast to compensable conditions (Jay et al. 2011; Gagnon et al. 2013; Cramer and Jay 2014), the present data indicate that it is not necessary to perform separate experiments with different exercise intensities for time‐dependent comparisons of core temperature and sweating responses during uncompensable heat stress. Fixing H_prod_ in either W·kg^−1^ or W·kg^−1^>E_max_ during uncompensable heat stress results in similar ΔT_re_ (Fig. 2) and LSR (Fig. 4) between groups vastly different in body size, however, a systematic difference in ΔT_sk_ existed with the former method (W·kg^−1^; Fig. 3). Biophysical influences may explain differences in core temperature during uncompensable heat stress previously ascribed to other factors. For example, sex‐related differences in core temperature have been reported with exercise at a %VO_2max_ (Horstman and Christensen 1982), however, the greater VO_2max_ in males compared to females would have resulted in a greater H_prod_ (in W·kg^−1^), which may have been responsible for the greater change in core temperature based on present findings. Furthermore, the present findings can potentially augment existing heat tolerance test protocols that employ a fixed treadmill walking speed on an incline (Moran et al. 2004; Druyan et al. 2013; Cheuvront 2014). While walking at the same speed and incline with a similar movement economy will lead to a similar W·kg^−1^ of H_prod_ between participants of different body masses, alterations in H_prod_ in W·kg^−1^ secondary to differences in walking efficiency would, according to the present observations (Fig. 2), result in systematic differences in ∆T_re_. Indeed, differences in walking efficiency of ~15–20% at a fixed speed/incline have been previously reported as a function of body size (Browning et al. 2006) and age (Malatesta et al. 2003), therefore, we recommend heat tolerance tests should be specifically conducted at a fixed H_prod_ in W·kg^−1^, verified with indirect calorimetry measurements, in order to ensure the endogenous heat stress relative to the biophysical characteristics of the participant are standardized and an unbiased comparison of core temperature responses can be achieved between different individuals.

Finally, it was assumed that all participants were unacclimated to the heat. It has been well demonstrated a defining characteristic of heat acclimation is an increased *ω*_max_ from ~85% to 100% (i.e., complete saturation of skin in sweat) (Candas et al. 1979b), while differences in aerobic fitness can also theoretically alter *ω*_max_ secondary to a partial heat acclimation. However, the measured E_max_ in W·m^−2^ (and therefore *ω*_max_; (Gagge 1937)) was similar between LG and SM groups (Table 2) despite a greater aerobic fitness in SM (Table 1) thereby suggesting a similar acclimation status between both groups independent of any fitness effect. While the greater aerobic fitness in SM did not appear to present any benefits from a core temperature or sweating perspective, their subjective tolerance to uncompensable heat stress (i.e., dropout rate due to volitional exhaustion) was better compared to LG individuals, as corroborated by others (McLellan 2001; Selkirk and McLellan 2001; McLellan et al. 2009).

## Conclusion

In conclusion, exercise prescribed as either a fixed H_prod_ of W·kg^−1^ or W·kg^−1^>E_max_ yielded similar changes in ΔT_re_ during uncompensable heat stress between groups differing greatly in body size; however, the former method (W·kg^−1^) demonstrated systematic differences in ΔT_sk_. Whole‐body sweat rate and LSR were similar between LG and SM groups at all exercise intensities suggesting that a maximum sudomotor output and a similar degree of uncompensability were attained in all trials. This study expands our previously developed methodological framework to higher levels of hyperthermia.

## Conflict of Interest

No conflicts of interest, financial or otherwise, are declared by any of the authors.

## Determining E_max_

*Step 1*. Upon arrival, height and weight must be measured initially to estimate body surface area (BSA) using the DuBois & DuBois equation (Druyan et al. 2013).

*Step 2*. Following instrumentation of skin and oesophageal temperature, and breath‐by‐breath recording of oxygen consumption (See Methods), have the participant exercise at a predetermined intensity in a hot (36°C) but dry (40% RH; 2.3 kPa) environment. It is best to select an intensity that a participant can sustain for the duration of the humidity ramp protocol (75 min). For example, this study utilized a fixed external work of 100W which was equal to ~50% of VO_2max_. Following 30 min of exercise where steady‐state core temperature and sweating are achieved and observed, humidity in the room will increase at a rate of 0.3 kPa (~5%RH) every 7.5 min in a stepwise fashion.

*Step 3*. The transition from a compensable to an uncompensable state is determined as immediate and upward rise in oesophageal temperature; exemplifying the biophysical inability to maintain heat balance. The absolute humidity coinciding with the upward rise in oesophageal temperature is confirmed using segmental regression (Cheuvront et al. 2009) where the 1‐minute averages of absolute oesophageal temperature are plotted against the respective ambient humidity. The absolute humidity at the intercept of each slope is defined as P_crit_.

*Step 4*. Using partitional calorimetry, one can then derive E_req_ for the minute of exercise coinciding with P_crit_ (Equation 1). E_req_, P_crit_, and P_sk,s_ (see Equation (4)) can then be substituted in to equation (7) to determine the individual's *K* coefficient.

*Step 5*. With the *K* coefficient, one can now derive the individual's actual E_max_ (in W·m^−2^) for the physical environment tested* by Equation (1). P_sk,s_ is estimated using Equation (4) and P_a_ is the humidity of the prospective experimental conditions.

*Ambient air flow, exercise modality, and clothing must remain identical to that of the E_max_ assessment trial to ensure accuracy of the estimated E_max_ in any subsequent experimental session.

## Determining net rate heat storage (W·kg^−1^ > E_max_):

In hot and humid conditions, the primary means for heat dissipation is the evaporation of sweat from the body surface, and thus the required heat loss can be defined as E_req_. By definition, E_req_ will exceed E_max_ during uncompensable heat stress. By knowing the E_max_ in a given condition, and estimating E_req_ using partitional calorimetry (Equation (1)), the difference will be the net rate of heat storage (in W·m^−2^). By further multiplying the net rate of heat storage by the participant's BSA, one can estimate the absolute net rate of heat storage, and this can be further expressed relative to the participant's mass (W·kg^−1^ > E_max_).
